# Catalpol relieved angiotensin II-induced blood–brain barrier destruction via inhibiting the TLR4 pathway in brain endothelial cells

**DOI:** 10.1080/13880209.2022.2142801

**Published:** 2022-11-12

**Authors:** Yu Xia, Yun Wei Lu, Ren Juan Hao, Gu Ran Yu

**Affiliations:** Department of Neurology, Jiangsu Province Hospital of Chinese Medicine, The Affiliated Hospital of Nanjing University of Chinese Medicine, Nanjing, China

**Keywords:** Hypertension, cerebral small vessel disease, inflammation, transcytosis, bEnd.3

## Abstract

**Context:**

Catalpol is a major bioactive constituent of *Rehmannia glutinosa* Libosch (Scrophulariaceae), a traditional Chinese medicine, which is widely used in multiple diseases, including hypertension.

**Objectives:**

To explore whether catalpol protects against angiotensin II (Ang II)-triggered blood–brain barrier (BBB) leakage.

**Materials and methods:**

The bEnd.3 cells and BBB models were pre-treated with or without catalpol (50, 200 and 500 μM) or TAK-242 (1 μM) for 2 h and then with Ang II (0.1 μM) or LPS (1 μg/mL) for 24 h. Cell viability was determined by the MTT assay. The levels of Toll-like receptor 4 (TLR4), myeloid differentiation factor 88 (MyD88), inducible nitric oxide synthase (iNOS), tumour necrosis factor-α (TNF-α), caveolin-1 (Cav-1) and p-eNOS/eNOS were tested by western blot. The BBB permeability was evaluated by the flux of bovine serum albumin-fluorescein isothiocyanate (BSA-FITC) across monolayers. nuclear factor kappa-B (NF-κB) p65 nuclear translocation was explored by immunofluorescence staining.

**Results:**

Ang II (0.1 μM) decreased the cell viability to 86.52 ± 1.79%, elevated the levels of TLR4, MyD88, iNOS, TNF-α and Cav-1 respectively to 3.7-, 1.5-, 2.3-, 2.2- and 2.7-fold, reduced the level of p-eNOS/eNOS to 1.6-fold in bEnd.3 cells, and eventually increased BBB permeability. Catalpol dose-dependently reversed these changes at 50–500 μM. Meanwhile, catalpol (500 μM) inhibited the upregulated levels of TLR4 pathway-related proteins and NF-κB p65 nuclear translocation, decreased the enhanced transcytosis, and relieved the BBB disruption caused by both LPS (the TLR4 activator) and Ang II. The effects are same as TAK-242 (the TLR4 inhibitor).

**Conclusions:**

Catalpol relieved the Ang II-induced BBB damage, which indicated catalpol has high potential for the treatment of hypertension-induced cerebral small vessel disease (cSVD).

## Introduction

Hypertension is one of the known causes and risk factors of cerebral small vessel disease (cSVD) which contributes to 45% of dementia cases (Cannistraro et al. 2019). Blood–brain barrier (BBB) disruption is a significant pathological feature of cSVD (Cuadrado-Godia et al. 2018). Dysfunction of BBB, the dynamic physical barrier which maintains the homeostasis of the microenvironment in the central nervous system (CNS), plays a crucial role in the pathogenesis of vascular cognitive impair (Abbott et al. 2010; Ueno et al. 2016). Angiotensin II (Ang II), a pivotal peptide of renin–angiotensin system (RAS), is involved in the regulation of blood pressure. In hypertension, elevated circulating levels of Ang II lead to destruction of the BBB integrity (Biancardi and Stern 2016). Therefore, any attempt at hypertension-induced cSVD should take the Ang II-triggered BBB leakage into account.

The properties of the BBB are mainly manifested within the endothelial cells (ECs). Since ECs in CNS have extremely low rates of transcytosis compared to peripheral ECs, movement between the brain and the blood can be tightly regulated (Daneman and Prat 2015). Ang II has proved to be important in the development of endothelial dysfunction (Radenkovic et al. 2016). In addition, researches both *in vivo* and *in vitro* reported that Ang II can enhance transcytosis in brain ECs through upregulating the expression of caveolin-1 (Cav-1) (Atış et al. 2019; Guo et al. 2019). Ang II is also recognized as a pivotal mediator in the processes of inflammation that result in cell injury (Benigni et al. 2010). All these contribute to Ang II-induced BBB disruption.

Toll-like receptor 4 (TLR4) is expressed in ECs abundantly and plays an important role in endothelial inflammation and endothelial dysfunction (Qu et al. 2020). Increasing evidence demonstrates that Ang II activates the proinflammatory responses partly by upregulating TLR4 expression and/or stimulating TLR4-dependent pathways in multiple cells (Biancardi et al. 2017). In addition, it has been reported TLR4 is involved in the regulation of the activity of Cav-1 (Mollace and Gliozzi 2016). Consequently, TLR4 might have an important role in Ang II-induced BBB dysfunction.

Catalpol, an iridoid glycoside, is one of the major bioactive constituents of the root of *Rehmannia glutinosa* Libosch (Scrophulariaceae) which is widely used in traditional Chinese medicine. Catalpol could transport into the brain rapidly after intravenous administration, which indicates that catalpol partially passes through the BBB (Xue et al. 2015). Catalpol has been extensively studied and exhibits multiple protective effects including neuroprotective, anti-inflammatory and antioxidant effects (Bhattamisra et al. 2019). Our past study showed that catalpol alleviated fibrillary Aβ_1–42_-induced BBB disruption through relieving ECs apoptosis, upregulating tight junction (TJ) scaffold proteins, LRP1 and P-gp, and downregulating expression of RAGE (Liu et al. 2018). Therefore, we considered that catalpol might have a potential protective effect on Ang-II mediated BBB damage.

In our study, we built a monolayer BBB model *in vitro* to investigate the effect of catalpol to Ang II-triggered BBB leakage. The roles of TLR4 activation on the Ang II-induced BBB disruption and the underlining mechanisms of the protective effects in catalpol on alleviating Ang II-triggered BBB leakage were further explored.

## Materials and methods

### Materials

Catalpol powder (purity >98%) was purchased from Chengdu ManSiTe Biotechnology Co. Ltd. (Chengdu, China). Ang II (purity ≥93%), 3-(4,5-dimethyl-2-thiazolyl)-2,5-diphenyl-2H-tetrazolium bromide (MTT), lipopolysaccharide (LPS) from *Escherichia coli* 0111: B4, and dimethyl sulphoxide (DMSO) were obtained from Sigma (St. Louis, MO). Resatorvid (TAK-242) was purchased from MedChemExpress (Monmouth Junction, NJ). Dulbecco’s modified Eagle’s medium (DMEM), phosphate-buffered saline (PBS, pH 7.4) and foetal bovine serum (FBS) were from GIBCO (Grand Island, NY). Bovine serum albumin-fluorescein isothiocyanate (BSA-FITC) was from Shanghai Solarbio Bioscience & Technology Co. Ltd. (Seebio Biotechnology, Shanghai, China). Protease inhibitor cocktail was from Beyotime (Beijing, China). Antibodies against TLR4, myeloid differentiation factor 88 (MyD88), tumour necrosis factor-α (TNF-α), nuclear factor kappa-B (NF-κB) p65, inducible nitric oxide synthase (iNOS), Cav-1 and β‐actin were from Proteintech (Chicago, IL). Antibodies against p-eNOS and eNOS were from Affinity Biosciences (Cincinnati, OH).

### Cell culture

bEnd.3 cells from ATCC (Manassas, VA) are immortalized mouse brain ECs. The 4th to 10th passages of cells in logarithmic growth phase were used in the following studies. bEnd.3 cells were cultured in DMEM containing 10% FBS, 100 ug/ml streptomycin, 100 U/mL penicillin and 100 mg/mL amphotericin in a 37 °C, 5% CO_2_/95% air incubator. The cell medium was renewed every two days.

### Drugs and treatment

Ang II was dissolved in PBS to a concentration of 10 mM and aliquots stored at −80 °C. Catalpol was dissolved in PBS to a concentration of 8 mM and aliquots stored at −80 °C. For groups treated with both Ang II/LPS and catalpol/TAK-242, catalpol or TAK-242 was added to cells 2 h prior to Ang II or LPS. After incubation with Ang II/LPS for 24 h, cells were collected for further analyses.

### Cell viability

The viability of bEnd.3 cells was determined by the MTT assay. bEnd.3 cells were seeded in 96-well plates at a density of 5000 cells per well and incubated for 24 h. Pre-treatment with or without catalpol (50, 200 and 500 μM) (Liu et al. 2018), cells were incubated with Ang II (0.1 μM) for 24 h. Then 20 μL of MTT (5 mg/mL) was added into each well and incubated for 4 h. The medium was discarded and 150 μL DMSO was added into each sample. Shaken for 10 min and a microplate reader (ELX-800, BIOTEK, Winooski, VT) was used to measure the absorbance of each sample at 490 nm.

### The BBB model *in vitro* and permeability of BBB measurement

Polyester transwell inserts (0.4 μM pore size, 6.5 mm diameter, 24 well, Nest, Wuxi, China) were coated with rat tail collagen (Corning, Corning, NY, 50 μg/mL in PBS), then bEnd.3 cells were seeded at a density of 50,000 cells/cm^2^ on the upper surface of the membrane. The culture medium was changed every two days. A week later, the medium was replenished every day. Millicell-ERS (Millipore, Burlington, MA) was used for testing the transendothelial electrical resistance (TEER) across bEnd.3 cells *in vitro* BBB model. Values of TEER were expressed as Ω × cm^2^. After 3 weeks of incubation, the TEER values reached a plateau. We selected the monolayers with resistance over 200 Ω to determine the BBB permeability (Lu et al. 2021).

The permeability of BBB *in vitro* was evaluated by detecting the flux of BSA-FITC across monolayers. BSA (mw ≈ 66 kDa) can be observed in endothelial vesicles, it is a marker of transendothelial permeability (Deli et al. 2005).

Pre-treatment with or without catalpol (50, 200 and 500 μM) or TAK-242 (1 μM) for 2 h, BBB models were incubated with Ang II (0.1 μM) or LPS (1 μg/mL) for 24 h. After drug treatment, cells incubated with 200 μL Hank’s liquid-containing BSA-FITC (50 μg/mL) on top chamber for 1 h. Hank’s liquid (1 mL) was added into the bottom chamber; 100 μL liquid was aspirated from the bottom chamber for next measurement. The fluorescence intensity was measured by a multifunctional microplate reader (Synergy HT, BIOTEK, USA) with excitation wave length at 485 ± 20 nm and emission wave length at 528 ± 20 nm.

The clearance volume (μL)=(*C*_A_×*V*_A_)/*C*_L_. *C*_A_ is the BSA-FITC concentration of the bottom chamber. *V*_A_ is the volume of the bottom chamber. *C*_L_ is the BSA-FITC concentration of the top chamber. Clearance rate (μL/min) is represented by the slope of clearance volume and time and can be shown as permeability (*P*)×surface area (*S*). The real permeability of BBB (*P*_e_) (μL/min/cm^2^) is calculated by this formula: 1/*PS*_e_=1/*PS*_t_ – 1/*PS*_i_. *PS*_t_ is the permeability of BBB in each experimental group (*P*_t_)×surface area (*S*). 1/*PS*_i_ is the permeability of BBB in the groups of the transwell inserts without cells (*P*_i_)×surface area (*S*) (Deli et al. 2005; *C*_L_=50 μg/mL, time = 60 min, *S* = 0.33 cm^2^, VA = 1000 μL, *C*_t_ is the value of the BSA-FITC concentration of the bottom chamber in each experimental group, *C*_i_ is the value of the BSA-FITC concentration of the bottom chamber in transwell inserts without cells)

The formula for calculating BBB permeability is:

$$P_{e}\;(\mu L/\min\cdot {\mathrm{cm}}^{2})\;=\;\frac{20\times C_{t}\times C_{i}}{19.8\times (C_{i}-C_{t})}$$

### Western blot analysis

Cells were seeded in six-well plates at a density of 15,000 cells/cm^2^ and treated as indicated. Cells were lysed in RIPA lysis buffer (Beyotime, Beijing, China) containing a protease inhibitor cocktail after being washed by ice-cold PBS. Lysates were centrifuged and collected. The concentration of protein was determined by BCA protein assay kit (Beyotime, Beijing, China). Protein (20 μg) was loaded each channel on sodium dodecyl sulphate-polyacrylamide gel electrophoresis (SDS-PAGE) gel, then transferred to polyvinylidene fluoride membranes. Following this, membranes were blocked in 5% non-fat milk in TBST (tris-buffered saline with 0.1% Tween 20), and then incubated with the primary antibodies overnight at 4 °C: TLR4 mouse monoclonal antibody (1:4000, Proteintech, Chicago, IL), iNOS rabbit polyclonal antibody (1:1000, Proteintech, Chicago, IL), MyD88 mouse monoclonal antibody (1:4000, Proteintech, Chicago, IL), TNF-α rabbit polyclonal antibody (1:1000, Proteintech, Chicago, IL), Cav-1 rabbit polyclonal antibody (1:2000, Proteintech, Chicago, IL), β-actin mouse monoclonal antibody (1:5000, Proteintech, Chicago, IL). After being washed three times with TBST, the membranes were incubated in the secondary antibodies (1:3000, Servicebio, Wuhan, China) at room temperature for 1 h. The blots were visualized in Gel Imager System (CHEMIDOC XRS+, BIO-RAD, Hercules, CA) using enhanced chemiluminescence (Biosharp, Shanghai, China). Image Lab software was used to quantify the band density.

### Immunofluorescence staining

The cells were seeded on coverslips in 12-well plates at a density of 15,000 cells/cm.^2^ Pre-treatment with or without catalpol (500 μM) or TAK-242 (1 μM), cells were incubated with Ang II (0.1 μM) or LPS (1 μg/mL) for 24 h. After being fixed with 4% paraformaldehyde for 20 min and permeabilized with 0.3% Triton X-100 for 30 min at room temperature, cells were incubated in immunol staining blocking buffer (Beyotime, Beijing, China) for 1 h. Primary antibody was used for overnight incubation: NF-κB p65 rabbit polyclonal antibody (1:500, Proteintech, Chicago, IL). After that, cells were incubated with fluorescent-labelled secondary antibody (1:1000, Proteintech, Chicago, IL) for 1 h in the dark at room temperature. 4′,6-diamidino-2-phenylindole (DAPI) was used to staining the nuclei for 10 min. Images were taken with a fluorescence microscope (DS-Qi2, NIKON, Tokyo, Japan) and semi-quantified by Image J.

### Endocytosis of BSA-FITC in bEnd.3 cells

The bEnd.3 cells were cultured on coverslips in 12-well plates at a density of 15,000 cells/cm^2^ and treated as indicated. Then, cells were incubated with BSA-FITC (50 μg/mL) in the dark at 37 °C for 1 h. After being washed with cold PBS, DAPI used to stain the nuclei for 1 min. Images were taken with a fluorescence microscope (DS-Qi2, NIKON, Tokyo, Japan) and each image was semi-quantified by Image J (Bethesda, MD).

### Statistical analysis

All experiments were replicated independently three times. All data were expressed as mean ± standard error and followed the law of normal distribution. Comparisons were performed by one-way analysis of variance (ANOVA) and followed by Tukey’s multiple comparison *post hoc* test. Statistical analysis was performed with SPSS 22.0 software (SPSS Inc., Chicago, IL). Differences between the groups were statistically significant at *p* < 0.05 for all tests.

## Results

### The expression of TLR4 in bEnd.3 cells treated with different concentrations of Ang II

**Figure 1.**
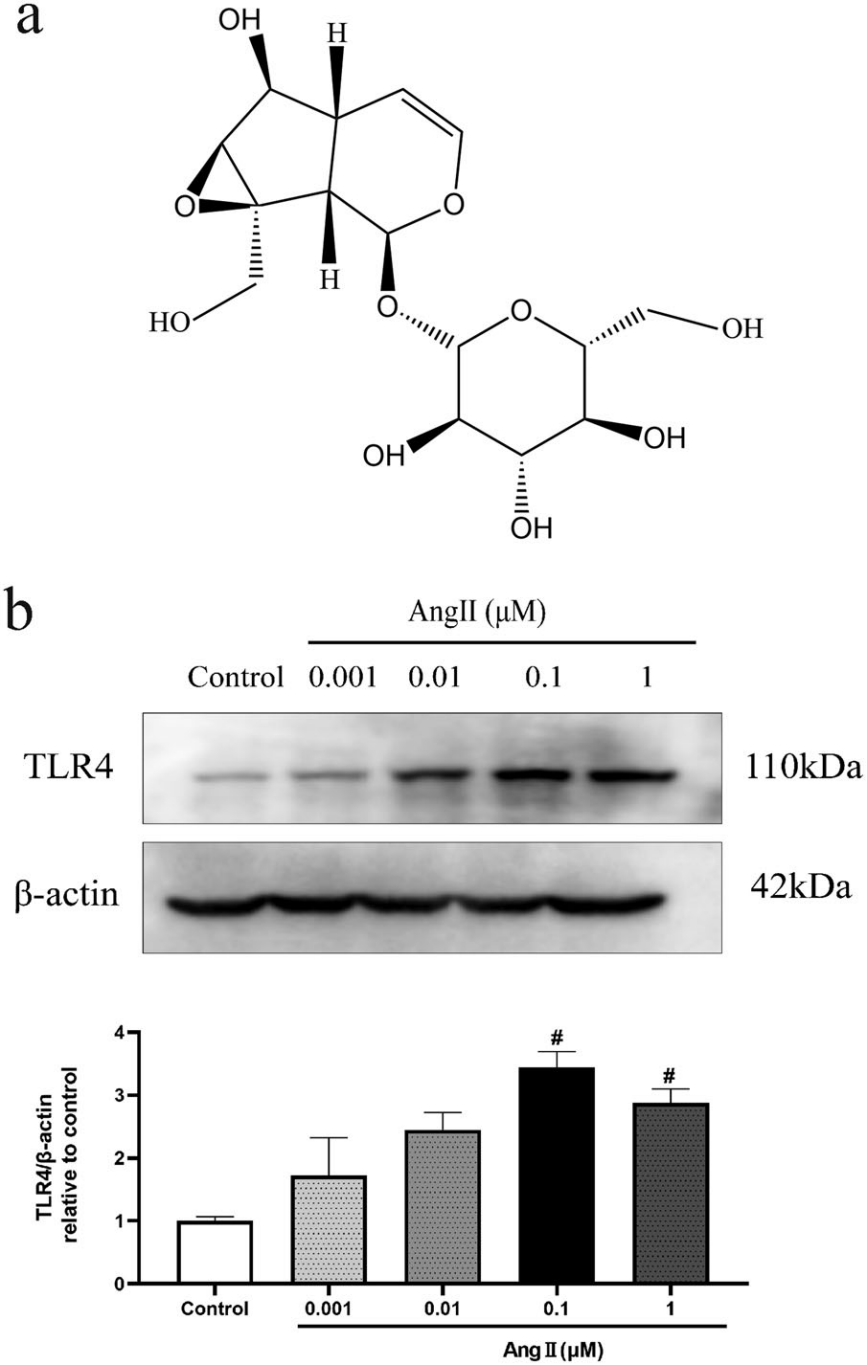
(a) Structure of catalpol. (b) Effect of different concentrations of Ang II (0.001, 0.01, 0.1 and 1 μM) on the expression of TLR4 in bEnd.3 cells, treatment for 24 h. The level of TLR4 was tested by western blot analysis. Actin levels were measured for the confirmation of equal amount of protein loading. All the results were represented as mean ± SEM from three independent experiments. ^#^*p*< 0.05 vs. control.

To explore the appropriate concentration of Ang II for this research, bEnd.3 cells were treated with different concentrations of Ang II (0, 0.001, 0.01, 0.1 and 1 μM) for 24 h. The level of TLR4 in bEnd.3 cells approached the maximum (3.4-fold increase) when exposed to 0.1 μM Ang II (Figure 1(b)). Therefore, 0.1 μM Ang II was chosen for subsequent experiments.

### Protective effect of catalpol against Ang II-induced cytotoxicity

**Figure 2.**
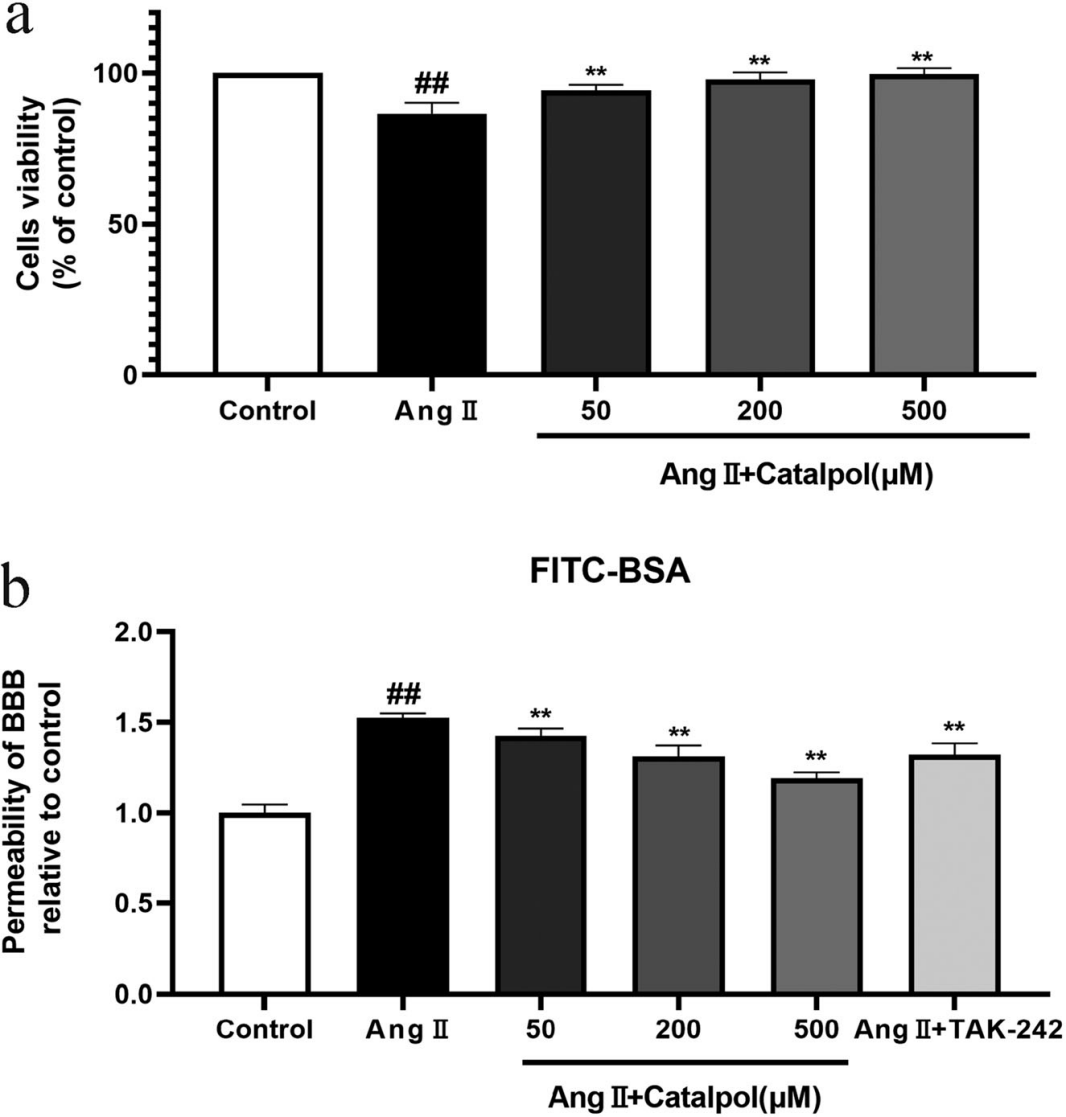
(a) Effect of catalpol (50, 200, 500 μM) on the cell viability of Ang II- treated bEnd.3 cells. The cell viability was measured by MTT assay. All the results were represented as mean ± SEM of the mean for six replicates from three independent experiments. (b) The permeability of in BBB models was represented by the calculated results of fluorescence intensity of FITC-BSA in the bottom chamber. All the results were represented as mean ± SEM from three independent experiments. ^##^*p* < 0.05 versus control. **p < 0.01 versus the group treated with Ang II.

In our past research, 0–500 μM catalpol showed no cytotoxicity in bEnd.3 cells (Liu et al. 2018). Exposed to 0.1 μM Ang II, the viability of bEnd.3 cells reached minimum (Lu et al. 2021). In this study, we observed the effects of different concentrations (50, 200 and 500 μM) of catalpol on the viability of 0.1 μM Ang II-treated bEnd.3 cells. In bEnd.3 cells incubated with Ang II, the cell viability decreased to 86.52 ± 1.79% of the control. After pre-treatment with 50, 200 and 500 μM catalpol, the cell viability increased to 94.25 ± 9.33%, 97.98 ± 1.12% and 99.60 ± 1.02% of the control. The results showed that catalpol increased the viability of Ang II-treated bEnd.3 cells (Figure 2(a)).

### Catalpol relieved BBB leakage induced by the Ang II

To determine the permeability of BBB *in vitro*, we built the mono-culture BBB models. BBB models treated with Ang II showed 1.5-fold higher flux of BSA-FITC across the mono-culture barrier layer compared to the control, which indicated the BBB destruction. Pre-treatment with catalpol (50, 200 and 500 μM) or TAK-242 (the TLR4 inhibitor, 1 μM) alleviated the Ang II-induced barrier permeability damage, of which 500 μM catalpol was the most effective (Figure 2(b)).

### Effects of catalpol on expression of the TLR4 pathway proteins in Ang II-stimulated bEnd.3 cells

**Figure 3.**
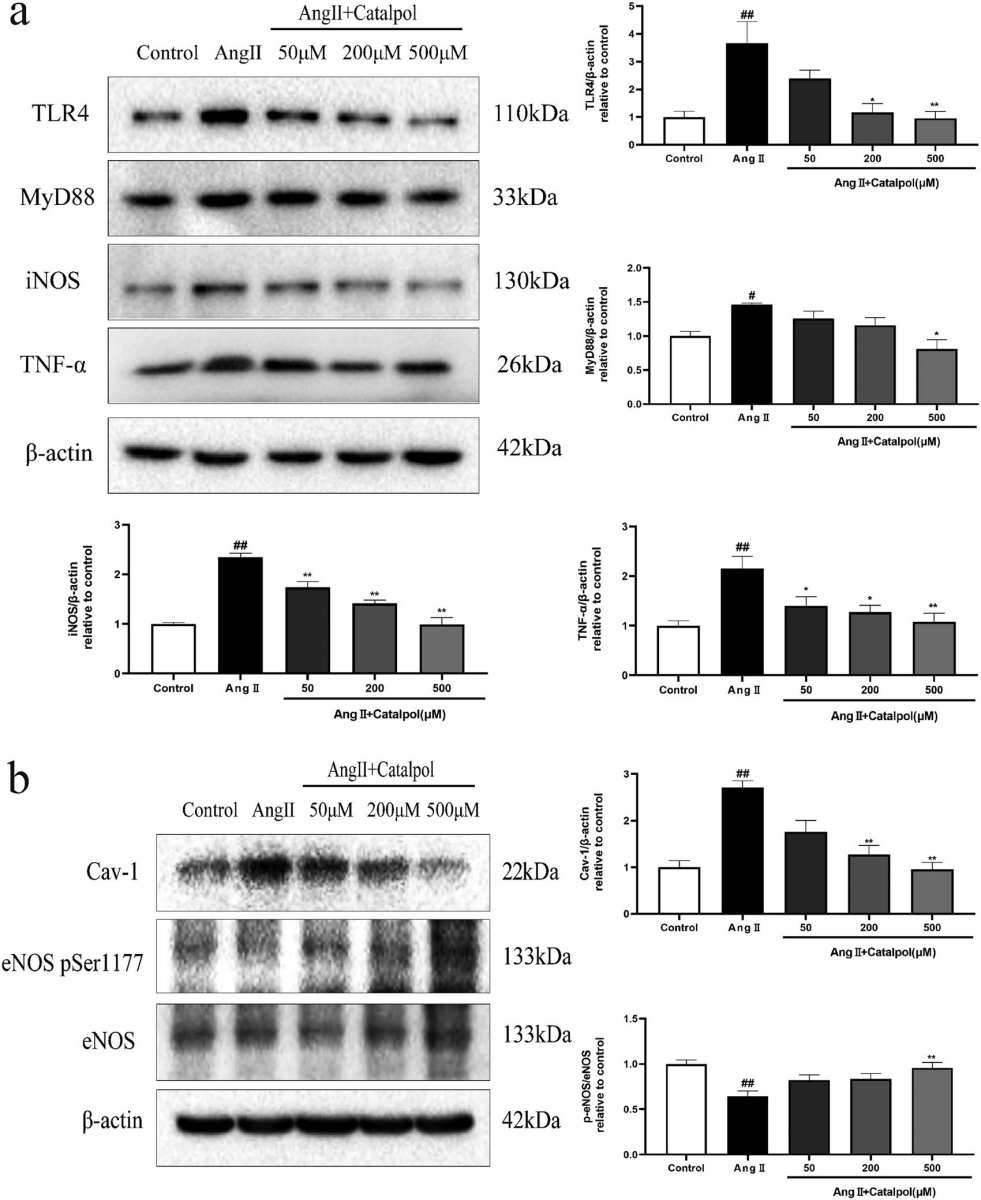
(a) The bEnd.3 cells were pre-treated with or without catalpol (50, 200 and 500 μM) for 2 h and then treated with Ang II (0.1 μM) for 24 h. Western blot analysis showed the effect of catalpol on the expression of TLR4, MyD88, iNOS and TNF-α in Ang II-treated bEnd.3 cells. (b) Effect of catalpol on the expression of Cav-1 and p-eNOS/eNOS in Ang II-treated bEnd.3 cells. Actin levels were measured for the confirmation of equal amount of protein loading. All the results were represented as mean ± SEM from three independent experiments. ^#^*p*< 0.05, ^##^*p*< 0.01 vs. control. *p< 0.05, **p< 0.01 vs. the group treated with Ang II.

Compared with the control group, the levels of TLR4, MyD88, iNOS, TNF-α and Cav-1 showed 3.7-, 1.5-, 2.3-, 2.2- and 2.7-fold increase in bEnd.3 cells incubated with 0.1 μM Ang II. The level of p-eNOS ser 1177/eNOS in Ang II-treated bEnd.3 cells showed a 1.6-fold reduction. The changes were reversed by pre-treatment with catalpol (50, 200 and 500 μM); 500 μM catalpol worked best (Figure 3(a,b)).

### Relationship between effects of catalpol on Ang II-induced BBB disruption and TLR4 signalling pathway

The above-mentioned results showed that catalpol may relieve Ang II-induced ECs injury and BBB damage and downregulate the levels of TLR4, MyD88, iNOS, TNF-α, Cav-1 and p-eNOS ser 1177/eNOS in bEnd.3 cells treated with Ang II. To evaluate whether catalpol inhibits the BBB damage through TLR4 pathway, bEnd.3 cells were pre-treated with or without catalpol (500 μM) or TAK-242 (TLR4 pathway inhibitor, 1 μM) for 2 h and then incubated with Ang II (0.1 μM) or LPS (TLR4 activator, 1 μg/mL) for 24 h.

**Figure 4.**
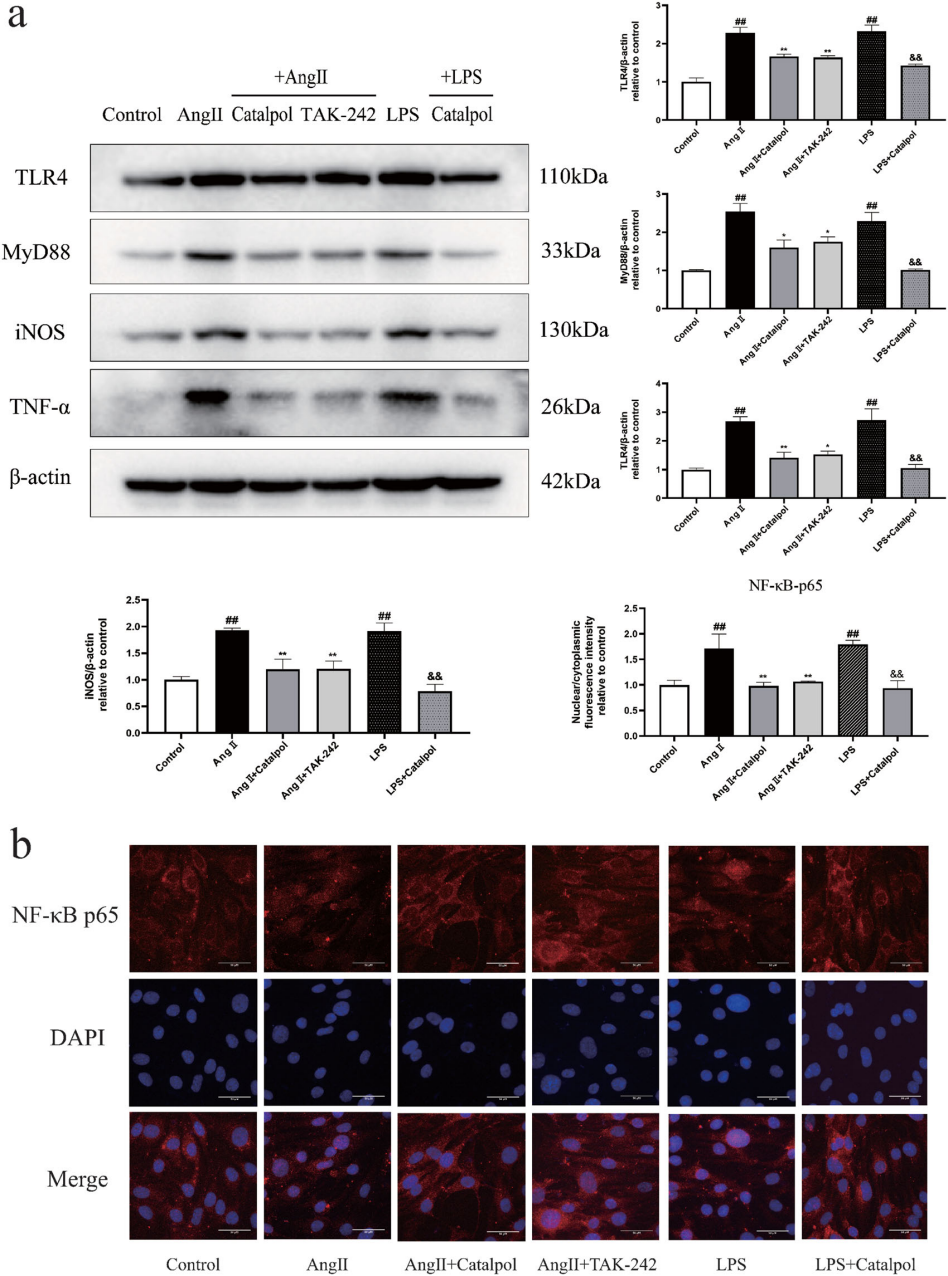
(a) Western blot analysis showed the levels of TLR4, MyD88, iNOS, TNF-α in Ang II-or LPS- stimulated bEnd.3 cells pretreated with or without catalpol or TLR4 pathway inhibitor TAK-242. Actin levels were measured for the confirmation of equal amount of protein loading. (b) Immunofluorescence staining showed the nuclear translocation of NF-κB p65 in bEnd.3 cells with different treatments. All the results were represented as mean ± SEM from three independent experiments. ^##^*p* < 0.01 versus control. *p < 0.05, **p < 0.01 versus the group treated with Ang II. ^&&^*p* < 0.01 versus the group treated with LPS.

**Figure 5.**
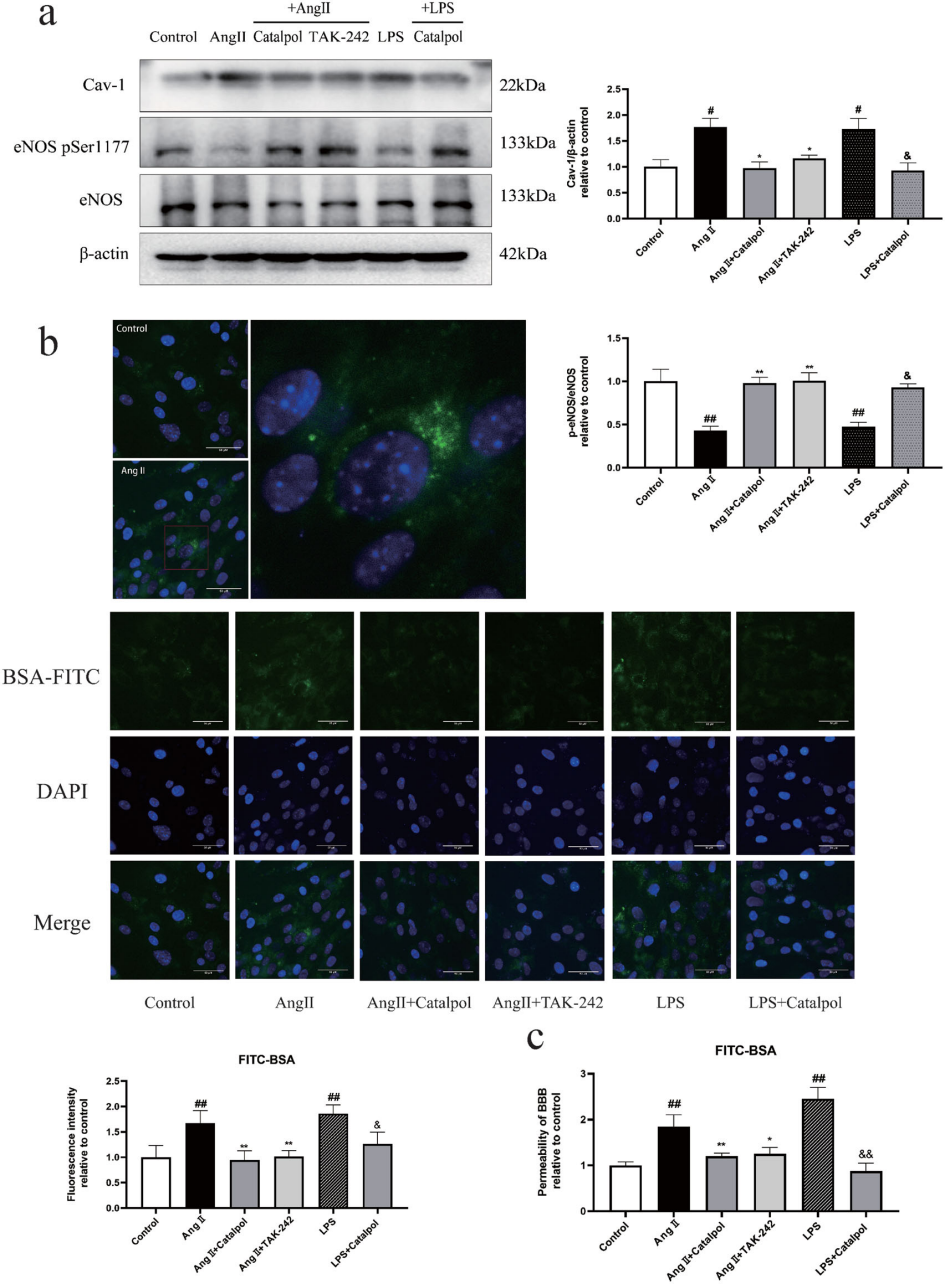
(a) Western blot analysis showed the levels of Cav-1 and p-eNOS/eNOS in 24 h Ang II (0.1 μM)- or LPS (1 μg/mL)-stimulated bEnd.3 cells pre-treated with or without catalpol (500 μM) or TAK-242 (1 μM). Actin levels were measured for the confirmation of equal amount of protein loading. (b) Endocytosis in bEnd.3 cells with the same treatment as (a) was detected by the fluorescent signals of the uptake of BSA tracers. Images were taken with a fluorescence microscope at a magnification of ×400. The fluorescence intensity was semi-quantified using Image J (Bethesda, MD). (c) Pre-treated the BBB models with or without catalpol (500 μM) or TAK-242 (1 μM) for 2 h and then added Ang II (0.1 μM) or LPS (1 μg/mL) into the BBB models for 24 h. The permeability in BBB models with different treatments was represented by the calculated results of fluorescence intensity of FITC-BSA in the bottom chamber. All the results were represented as mean ± SEM from three independent experiments. ^#^*p*< 0.05, ^##^*p*< 0.01 vs. control. *p< 0.05, **p< 0.01 vs. the group treated with Ang II. ^&^*p*< 0.05, ^&&^*p*< 0.01 vs. the group treated with LPS.

Treatment with Ang II or LPS significantly increased the levels of TLR4, MyD88, iNOS, TNF-α, Cav-1 and p-eNOS ser 1177/eNOS compared with the control. Ang II or LPS stimulation also promoted nuclear translocation of the NF-κB p65. Pre-treatment with catalpol or TAK-242 downregulated the levels of these proteins (Figures 4(a) and 5(a)) and blocked the translocation of the NF-κB p65 (Figure 4(a)) in Ang II- or LPS-treated bEnd.3 cells.

Subsequently, we tested the fluorescent signals of BSA-FITC in cells to detect the transcytosis in bEnd.3 cells. Ang II or LPS obviously increased the uptake of BSA tracers in bEnd.3 cells. Pre-treated with catalpol or TAK-242, the fluorescent intensity in bEnd.3 cells was significantly attenuated (Figure 5(b)).

We went further to determine whether the catalpol could relieve Ang II-mediated BBB destruction in the mono-culture BBB models through TLR4 pathway. Ang II or LPS significantly increased the flux of BSA-FITC across the mono-culture barrier layer compared with the control, which revealed the disruption of BBB models *in vitro*. Pre-treatment with catalpol or TAK-242 relieved Ang II- or LPS-induced BBB permeability decline (Figure 5(c)).

## Discussion

Ang II has been reported to modulate BBB permeability (Fleegal-DeMotta et al. 2009). Since BBB dysfunction is known to be crucial in the pathogenesis of cSVD (Wong et al. 2019), Ang II-mediated BBB destruction might play an important role in hypertension-induced cSVD. Therefore, alleviation of Ang II-triggered BBB dysfunction should be a notable strategy for the therapy of hypertension-induced cSVD. In this study, we demonstrated that catalpol relieved Ang II-induced BBB leakage through reducing inflammation, transcytosis and endothelial dysfunction in the bEnd.3 cells. The underlying mechanisms may rely on the inhibition of TLR4 signalling pathway in brain ECs.

The active family of Toll-like receptors (TLRs) initiates a series of downstream signalling cascades and then induces an inflammatory response and subsequently specific response through adaptor proteins (Akira and Takeda 2004). TLR4 is the most well defined TLR in the pathogenesis of hypertension. In hypertension, an interplay between Ang II and TLR4 triggers multiple effects including the vasculature, the kidneys and the CNS (Biancardi et al. 2017). In our research, Ang II obviously increased the BBB permeability. It also upregulated the expression of TLR4, MyD88 and induced the translocation of the NF-κB p65 which suggested the activating effect of Ang II on TLR4 pathway. Then, the expression of iNOS and TNF-α upregulated subsequently indicated the inflammation injury occurred in ECs. In addition, levels of Cav-1 were elevated and the phosphorylation of eNOS on Ser1177 was suppressed by Ang II, showed the enhanced transcytosis and endothelial dysfunction triggered by Ang II in the brain ECs. All the changes in Ang II-stimulated bEnd.3 cells were reversed by TAK-242, a specific inhibitor of TLR4. These results further demonstrated that Ang II can induce brain endothelial injury and dysfunction and then result in BBB damage eventually through activating the TLR4 signalling pathway.

Many studies indicated that some of the protective effects of catalpol are related to its regulation of the TLR4 pathway. Catalpol prevented spinal cord injury by relief of oxidative stress and inflammation via upregulation of miR-142, which downregulated the expression of HGMB1 to restrain the activation of TLR4/NF-κB (Xia et al. 2020). Catalpol also suppressed LPS-mediated neuro-inflammation in BV2 microglia through antagonizing the TLR4/NF-κB signalling pathway (Choi 2019). In addition, catalpol has been shown to ameliorate apoptosis and inflammation in high glucose-damaged podocytes through inhibiting NOX4 and suppressing the TLR4/MyD88 and p38 MAPK signalling pathways (Chen et al. 2019). Past research revealed that catalpol can directly bind to TLR-4 and Src to alleviate the microvascular barrier damage and haemorrhage triggered by LPS (Zhang et al. 2016). Collectively, all the studies suggested that TLR4 plays an important role in the protective effect of catalpol. In this research, pre-treatment of catalpol decreased the expression of TLR4 in Ang II-injured ECs. In the LPS- (the activator of TLR4) treated bEnd.3 cells, catalpol showed the same effect. The results indicated catalpol can restrain the activity of TLR4 in ECs stimulated by Ang II.

Upon activation of TLR4 in the MyD88-dependent pathway, NF-κB translocates into the nucleus after a series of downstream signalling to regulate inflammatory cytokine production, such as TNF-α, iNOS, IL-1β, IL-6, etc. (Liu et al. 2014). MyD88 is crucial for the production of inflammatory cytokines induced by all TLRs, including TLR4 (Takeda and Akira 2004). Overproduction of these inflammatory mediators is related to neuroinflammation (Leitner et al. 2019), as well as ECs injury and dysfunction (Sprague and Khalil 2009). Targeting the TLR4 signalling pathway is considered as an important therapeutic strategy for neuroinflammation (Rahimifard et al. 2017). In our study, pre-treatment of catalpol reduced the increased expression of MyD88, TNF-α and iNOS and the nuclear translocation of NF-κB in Ang II-treated ECs, to the same level as TAK-242. Catalpol also antagonized LPS-induced TLR4 pathway activation and the subsequent increased level of related protein and the enhanced nuclear translocation of NF-κB. These results proved that catalpol reduced the generation of inflammatory cytokines in Ang II-treated bEnd.3 cells through the TLR4/MyD88 pathway. This may relieve the consequent injury and dysfunction of brain ECs mediated by Ang II.

Endothelial cells express a high level of caveolae and Cav-1 (Xu et al. 2015). Cav-1 is the principal structural and signalling component of caveolae, the site of the vesicular trafficking in cells (Mollace and Gliozzi 2016). High expression of Cav-1 results in enhanced transcytosis, and then increased the BBB permeability (Soares et al. 2014). Besides, there is evidence that caveolae may reduce the TJ proteins (Xu et al. 2015). In studies both *in vivo* and *in vitro*, Ang II increased the expression of Cav-1 and eventually led to the dysfunction of the BBB (Atış et al. 2019; Guo et al. 2019). It has been reported that TLR4 highly regulates the activity of Cav-1 (Jiang et al. 2014; Mollace and Gliozzi 2016). In this study, catalpol downregulated the level of Cav-1 and decreased the transcytosis in Ang II-treated brain ECs, to a similar level as TAK-242. Catalpol also decreased the expression of Cav-1 and transcytosis in cells that activated the TLR4 signalling pathway with LPS. Our result revealed that catalpol may reduce the enhanced transcytosis through inhibiting the TLR4 signalling pathway to exert the protective effect on Ang II-mediated BBB disruption. eNOS is a master regulator in endothelial function through the generation of nitric oxide (NO), and acts as an essential role in regulating vascular tone and homeostasis (Siragusa and Fleming 2016). Phosphorylation of Ser 1177 results is an important mechanism in upregulating eNOS activity (Ali et al. 2018). Previous study suggested that Ang II activates protein phosphatase 2A (PP2A) leading to the inhibition of eNOS Ser1177 phosphorylation, and eventually results in endothelial dysfunction (Luo et al. 2019). eNOS is localized on caveolae, and binding with Cav-1 suppresses its activity (Oliveira and Minshall 2018). Therefore, the upregulation of CAV-1 was proposed to inhibit the activation of eNOS (Kamoun et al. 2006). In our study, Ang II suppressed the activation of eNOS which may be related to the upregulated of Cav-1. iNOS is a metabolic enzyme under stress conditions which produces large, unregulated quantities of NO. iNOS-derived NO is associated with cell injury (Anavi and Tirosh 2020). The upregulation of iNOS and the inhibited activity of eNOS represent a favourable balance which positively improves vascular dysfunction (Silva et al. 2016). In our study, Ang II resulted in the imbalance of iNOS/eNOS that leads to the endothelial dysfunction. Pre-treatment with catalpol significantly increased the phosphorylation of eNOS on Ser1177 in bEnd.3 cells incubated with Ang II, to the same degree as TAK-242. Catalpol exerted the same effect in the LPS-treated cells. As mentioned above, catalpol reduced the level of iNOS by inhibiting the TLR4/MyD88 pathway. Combined with this result, it can be suggested that catalpol can normalize eNOS/iNOS imbalance in Ang II-treated brain ECs via the depression of TLR4 signalling. This is undoubtedly a further improvement of endothelial dysfunction caused by Ang II.

Since the properties of the BBB are mainly reflected in the brain ECs, a pathological state of ECs ultimately leads to BBB disruption. Inflammatory injury, enhanced transcytosis, and endothelial dysfunction are the changes in brain ECs due to Ang II-induced TLR4 activation. Catalpol manifested the protective effect against these pathologic changes in ECs via inhibiting TLR4 activity, and eventually relieved the BBB destruction triggered by Ang II.

However, this research has some limitations. Activation of TLR4 is not the single factor of Ang II-induced BBB disruption, TJ destruction and oxidative stress are involved in. In addition, treatment of ECs and BBB models with Ang II could not completely simulate the pathological changes of hypertension-induced cSVD. Therefore, we will continue to investigate the effects of catalpol on Ang II-induced BBB destruction in different aspects. At the same time, experiments *in vivo* will be improved to verify the protective effect of catalpol on hypertensive cSVD.

## Conclusions

In our study, Ang II triggered the inflammatory injury, enhanced transcytosis and eNOS/iNOS imbalance-induced endothelial dysfunction in ECs through activating the TLR4 signalling pathway and then led to BBB disruption. Catalpol relieved the Ang II-induced BBB damage and reversed all the changes in bEnd.3 cells treated with Ang II via inhibiting the TLR4 pathway. Our study revealed the protective effect of catalpol on Ang II-mediated BBB damage. These results provide evidence for the treatment of hypertensive cSVD with catalpol. In addition, it also proved that catalpol has significant potential for the development of new natural drugs for the treatment of hypertension-induced cSVD.
